# Pulmonary Tuberculosis Caused by *Mycobacterium bovis* in China

**DOI:** 10.1038/srep08538

**Published:** 2015-03-04

**Authors:** Guanglu Jiang, Guirong Wang, Suting Chen, Xia Yu, Xiaobo Wang, Liping Zhao, Yifeng Ma, Lingling Dong, Hairong Huang

**Affiliations:** 1 National Tuberculosis Clinical Laboratory, Beijing Key laboratory on Drug-resistant Tuberculosis Research, Beijing Chest Hospital, Capital Medical University, Beijing Tuberculosis and Thoracic Tumor Institute, Beijing 101149, China

## Abstract

The epidemiology of *Mycobacterium bovis* infection in humans in China is unknown. In this study, pulmonary tuberculosis caused by *M. bovis* in China was studied. A total of 4069 clinical strains isolated from sputa during the 2007–2009 nationwide surveillance of drug-resistant tuberculosis in China were analyzed. *M. bovis* was identified by para-nitrobenzoic acid and thiophen-2-carboxylic acid hydrazide growth tests, spoligotyping and multiplex PCR amplification. In addition, a total of 1828 clinical specimens were recruited from Beijing Chest Hospital (Beijing, China) for Löwenstein-Jensen (LJ) culture, both on standard LJ medium and LJ medium containing 4.5 mg/ml(W/V) sodium pyruvate, the latter being the preferred medium for *M. bovis* growth. The isolates which demonstrated more vigorous on pyruvate containing medium than on standard LJ medium were then identified by multiplex PCR amplification. Only 1 isolate from the nationwide surveillance was confirmed as *M. bovis*-BCG. The isolate belonged to a predominant spoligotype SB0120 (ST482). In addition, no *M. bovis* isolate was acquired by the continuous screening step in Beijing Chest Hospital. *M. bovis* has a negligible contribution to pulmonary tuberculosis in China, so neither laboratory identification nor clinical treatment of *M. bovis* infection need be considered in routine work.

Infection of humans with any organism of the *Mycobacterium tuberculosis* complex (MTC) can result in the disease known as ‘tuberculosis' (TB). Members of the MTC are highly related mycobacteria which share a high degree of homology at the genomic sequence level despite varying in pathogenicity, geographic range, certain physiological features, epidemiology, and host preference^1^. TB caused by *M. bovis* or *M. tuberculosis* cannot be distinguished clinically, radiologically, or pathologically in individual patients^2^. Since most laboratories do not fully identify MTC isolates into species, the true cause of TB in those patients and its source often remain undiscovered.

*M. bovis* is naturally resistant to pyrazinamide (PZA)^3^,^4^ which is classified as a first-line anti-tuberculosis drug. Unlike conventional antibiotics that are active mainly against growing bacteria, PZA has been reported to kill at least 95% of the semidormant population of *M. tuberculosis* that persists in acidic pH environments inside macrophages^5^. Therefore, PZA plays a unique role in shortening the duration of TB chemotherapy. The common side effects due to PZA are hyperuricemia, hepatotoxicity, dysuria, arthralgia, and sideroblastic anemia^6^. Therefore, due to the intrinsic resistance to PZA of *M. bovis*, complete identification of MTC isolates to the species level is required for epidemiology and for appropriate patient treatment and public health measures.

As the situation of *M. bovis* infection in China is not well understood, the objective of this study was to determine the situation of human pulmonary tuberculosis infected by *M. bovis* in China.

## Results

### *M. bovis* strain identification

A total of 245 out of the 4069 clinical isolates analyzed were susceptible to both PNB and TCH. Only 1 out of the 245 isolates was identified as *M. bovis* firstly by spoligotyping, whereas the multiplex PCR produced consistent outcomes but the only *M. bovis* isolate was identified as *M. bovis*-BCG. All the other 244 PNB-TCH sensitive strains were finally identified as *M. tuberculosis* by both methods.

### Case finding

The single *M. bovis*-BCG isolate was from a 33 year old male, who lived in Hebei province, which is a non-livestock-producing region located in northern China. The patient worked in a commercial company which was not involved with livestock. He did not have any known immune suppressed disease such as diabetes mellitus, and he was HIV negative. The patient had never previously been diagnosed with tuberculosis and there was no known tuberculosis case among his family members or friends. The patient reported cough and expectorate for less than 3 weeks before he consulted a doctor. After being diagnosed as pulmonary tuberculosis, he was administered HRZE2/HR4 regimen and was cured. The disease had not relapsed within the past 5 years.

### Molecular typing of the *M. bovis* isolate

The isolated *M. bovis* strain had the typical spoligotype profile of *M. bovis* with the absence of spacers 39–43 and the presence of the spacers 33–38^1^. The isolate belonged to a predominant spoligotype named SB0120 (ST482), with the corresponding hexacode of 6F-5F-5F-7F-FF-60.

**Figure 1:**
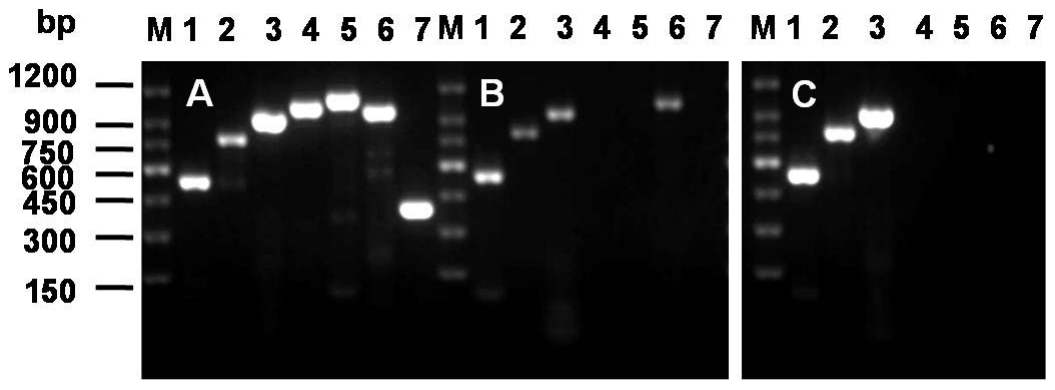
Multiplex PCR typing of mycobacteria. (A) *M. tuberculosis* H37Rv (ATCC 27294); (B) *M. bovis* (ATCC 19210); (C) our isolate. The PCR products were visualized by agarose gel electrophoresis and ethidium bromide staining. M represents 150 bp DNA ladder. Lanes: 1, 16S rRNA; 2, RV0577; 3, IS1561; 4, Rv1510; 5, Rv1970; 6, Rv3877/8; 7, Rv3120.

The same isolate had the representative multiplex PCR amplification pattern for *M. bovis*-BCG^1^ with the presence of 16S rRNA gene (543 bp), Rv0577 gene (786 bp) and IS1561 element (943 bp), but the absence of Rv1510 gene (1033 bp), Rv1970 gene (1116 bp), Rv3877/8 gene (999 bp)and Rv3120 gene (404 bp) (Figure 1).

### Sodium pyruvate containing LJ media culture screening and multiplex PCR identification

1828 sputum specimens were collected during the 3 months recruitment, and 548 specimens yielded positive culture outcomes. Among all the isolates, 43 isolates had more vigorous growth on sodium pyruvate containing LJ media than on standard LJ media and 12 isolates grew only on sodium pyruvate containing LJ media but not on the standard LJ media. All the 55 isolates were investigated by the multiplex PCR amplification but none were identified as *M. bovis*.

## Discussion

Before spoligotyping technique was introduced to differentiate *M. bovis* from *M. tuberculosis*, phenotypic assays including the TCH inhibition test were used as the major method for differentiation. The phenotypic assays are technically demanding and time-consuming, while spoligotyping has proved to be a more accurate alternative tool for *M. bovis* identification^7^. However, spoligotyping is technically complex, laborious, and costly which prevent it being applied in laboratories with limited resources. An alternative molecular method, multiplex PCR was introduced in 2003, and later use proved that it was very reliable in *M. bovis* identification^8^,^9^.

Between 2007 and 2009, the national surveillance on drug resistant TB was conducted in China, and 4069 isolates were obtained in the survey^10^. Since TCH sensitivity is an important bacteriological characteristic of *M. bovis*^11^, a gross identification was performed by doing TCH growth test, to screen for possible *M. bovis* isolates. A PNB growth test was also conducted to exclude interference from non-tubercular mycobacteria isolates. Among the 4069 clinical strains collected during the surveillance, 245 MTC strains sensitive to 5 μg/ml TCH were acquired. Further identification by spoligotyping and multiplex PCR only identified one *M. bovis*-BCG strain. This result was much lower than what has been reported in the other industrialized countries (0.5–7.2%)^12^. By contrast, developing countries are short of related data^15^ and some papers have speculated that *M. bovis* could account for as many as 10–15% of the estimated 7–10 million new cases of human TB occurring in those countries^11^,^12^,^13^. Previously, in China, there have been four national epidemiological surveys on human pulmonary tuberculosis in 1979, 1985, 1990 and 2010, but no data was available on *M. bovis* infection. However, the national surveys of cows, conducted in 1985 and 1987, showed the prevalence rates of bovine TB among cattle to be 5.83% and 5.43%, respectively^14^. Two localized studies from China also found *M. bovis* infection in human was very rare^15^,^16^. Chen et al^15^. reported 0.34%,(17/5011) of the human *M. bovis* infection among the hospitalized TB patients in an agricultural area of central China, and Taxitimemuer et al^16^. identified 1 *M. bovis* strain from 313 clinical isolates originated from sputum samples of pulmonary tuberculosis patients in Xinjiang autonomous region, which is the major pasture area in China. All those outcomes showed that *M. bovis* was seldom isolated among pulmonary tuberculosis patients in China. A similar situation of absence of *M. bovis* infection in humans was observed in Brazil, where not a single case of *M. bovis*-related TB was evidenced from 702 TB patients^17^. It is known that bovine TB could be transmitted between cows and humans through consumption of contaminated unpasteurized dairy products and raw or undercooked meat and through close contact with infected animals or their carcasses^18^. Human-to-human transmission can also occur although it remains rare^19^. In our study the fact that we did not identify one single *M. bovis* strain suggesting that the observed incidence of TB in cattle is not affecting the rates of pulmonary TB in the human population. The low risk to human health currently posed by *M. bovis* in China may be due to a combination of control measures: a national immunization program with BCG vaccine (an attenuated variant of *M. bovis*) administered to new-borns; pasteurization of milk; routine ante and post mortem inspection of cattle and other food-producing species at abattoirs; and a series of regulations and laws were enacted to control the prevalence of bovine TB. Recent report described that *M. tuberculosis* accounted for partial cattle infections in China^15^, but its influence on the human TB epidemiology in China is difficult to be evaluated.

During the surveillance, the sputum samples were cultured using standard LJ medium, which is optimal for *M. tuberculosis* growth but sub-optimal for *M. bovis* which prefers sodium pyruvate in the medium^20^. In order to exclude this as a factor in the low rate of isolation of *M. bovis*, we investigated 1828 sputum specimens collected from TB suspects in Beijing Chest Hospital by both standard LJ media and sodium pyruvate containing LJ media. However, no *M. bovis* strains were isolated by this step. Beijing Chest Hospital is a well known designated hospital for TB treatment in China, and about 70% of patients are not from local, but come from all over the country. This suggests that the incidence of *M. bovis* infection in humans in China as a whole is very low. In our random screening assay, some *M. tuberculosis* isolates yielded more colonies on sodium pyruvate LJ media than on glycerol LJ media, and some isolates only grew on sodium pyruvate LJ media, whereas those phenomena are beyond a reasonable explanation.

Something very unusual is that the *M. bovis*-BCG stain was cultured from an immune competent male in his productive years. Whether it is because the BCG strain harbors more virulence than usual is still not known, whole genome sequencing of this strain is currently being carried out to decipher this.

## Conclusion

There was no pulmonary TB infected by *M. bovis* in a national surveillance in China. This suggests that zoonotic TB has a low incidence and that TB caused by *M. bovis* poses a negligible threat to public health in China, and neither laboratory identification nor clinical treatment of *M. bovis* infection need be considered in routine work.

## Methods

### Ethics Statement

This study was approved by ethics committee of Beijing Chest Hospital, and the methods were carried out in accordance with the approved guidelines.

### Strains

A total of 4069 clinical strains isolated during the 2007–2009 nationwide survey on drug-resistant tuberculosis in China were analyzed in this study^10^. Written informed consent for participation was obtained from all the patients in the epidemiological study. The strains were collected from 70 centers around the country with at least one cluster in each of the 31 provinces and municipalities of China. The number of centers assigned to each province and municipality was proportional to the number of new smear-positive cases reported by that province relative to the total number of cases nationwide previously.

### Phenotypic screening

All of the isolates were screened by growth tests on LJ media containing 500 μg/ml para- nitrobenzoic acid (PNB) and 5 μg/ml thiophen-2-carboxylic acid hydrazide (TCH). Under these conditions, the classical *M. tuberculosis* strain which grows on TCH but not on PNB, can be differentiated from Asian strains of *M. tuberculosis*, *M. africanum* and *M. bovis*, which grow on neither one^21^. Strains sensitive to both PNB and TCH were further analyzed by spoligotyping and multiplex PCR amplification. The reference strains *M. tuberculosis* H37Rv ATCC 27294 and *M. bovis* ATCC 19210 were used as controls.

### Spacer oligonucleotide typing (spoligotyping)

Genomic DNA was extracted by boiling the freshly cultured bacteria. The PCR reaction and hybridization process was performed as described previously^7^. A commercially available kit (Isogen Bioscience BV, Maarssen; The Netherlands) was used as described by the manufacturer. The hybridization patterns were converted into binary formats and compared with previously reported *M. bovis* strains present in the spoligotype database (www.mbovis.org) and the global spoligotype database SpolDB4 (http://www.pasteur-guadeloupe.fr/tb/spoldb4). Quality control was performed using reference strains *M. tuberculosis* H37Rv ATCC 27294, and *M. bovis* ATCC 19210 as positive controls, using sterile deionised water as negative control.

### Multiplex PCR

The multiplex PCR was conducted to differentiate *M. tuberculosis* from *M. bovis* strains as previously described^1^. Seven sets of primers specific to 16S rRNA, Rv0577, IS1561 (Rv3349c), Rv1510 (RD4), Rv1970 (RD7), Rv3877/8 (RD1), Rv3120 (RD12) in *M. tuberculosis* H37Rv genome were synthesized by Shanghai Sangon Biological Technology and Services Co., Ltd., China. The isolates were defined as *M. tuberculosis* when PCR analysis yielded products from all seven sets of primers; as *M. bovis* when PCR analysis yielded products from 4 sets of primers, 16S rRNA, RV0577, IS1561, and Rv3877/8; as *M. bovis*-BCG when PCR analysis yielded products from only 3 sets of primers, 16S rRNA, RV0577 and IS1561. The reference strains *M. tuberculosis* H37Rv ATCC 27294 and *M. bovis* ATCC 19210 were used as controls.

### Sodium pyruvate containing LJ media culture screening

Consecutive clinical sputum specimens were recruited from Beijing Chest Hospital from May to July 2012, and duplicate samples from the same patients were excluded. The sputa were processed following the standard LJ medium culture protocol^21^. Briefly, the sputum was mixed with one volume of 4% sodium hydroxide and then homogenized by vigorous stirring. An aliquot of 0.1 ml of the mixture was inoculated onto both standard LJ medium containing glycerol and modified LJ medium containing pyruvate before incubation at 37°C. The standard LJ media were prepared following the WHO guidelines, while the pyruvate containing media were made by the same protocol but substituted the glycerol gradient with 4.5 mg/ml (w/v) sodium pyruvate^22^. Those isolates which demonstrated more vigorous on sodium pyruvate containing media or those isolates growing only on sodium pyruvate containing media were investigated further by multiplex PCR.

## Author Contributions

H.H. developed the study concept and protocol and all other authors reviewed the protocol and made contributions to study design. G.J. and G.W. participated in the acquisition of data and analysis and interpretation of the data. H.H. and G.W. drafted the manuscript. S.C., X.Y., X.W., L.Z., Y.M. and L.D. were involved in revising it critically for important intellectual content and have given final approval of the version submitted.
